# Immunohistochemical and molecular profiling of CD 117, Oct-4, and Sox-2 in canine cutaneous mast cell tumor of the crossbred dogs in Faculty of Veterinary Science, Chulalongkorn University, Bangkok, Thailand

**DOI:** 10.14202/vetworld.2021.2646-2654

**Published:** 2021-10-15

**Authors:** Sirilak Meesuwan, Dettachai Ketpun, Prapruddee Piyaviriyakul, Kasem Rattanapinyopituk, Pattharakrit Theewasutrakul, Achariya Sailasuta

**Affiliations:** 1 Veterinary Pathobiology Program, Graduate School, Department of Pathology, Faculty of Veterinary Science, Chulalongkorn University, Bangkok, 10330 Thailand; 2 Companion Animal Cancer Research Unit, CAC-RU, Faculty of Veterinary Science, Chulalongkorn University, Bangkok 10330, Thailand; 3 Veterinary Pathology and Diagnosis Centre, Akkhraratchakumari Veterinary College, Walailak University, Nakhon Si Thammarat 80160, Thailand; 4 One Health Research Centre, Walailak University, Nakhon Si Thammarat 80160, Thailand; 5 Department of Physiology, Biochemistry Unit, Faculty of Veterinary Science, Chulalongkorn University, Bangkok, 10330, Thailand; 6 Department of Pathology, Faculty of Veterinary Science, Chulalongkorn University, Bangkok, 10330, Thailand; 7 Oncology Clinic, Small Animal Teaching Hospital, Faculty of Veterinary Science, Chulalongkorn University, Bangkok 10330, Thailand.

**Keywords:** canine, CD 117, crossbred, mast cell tumor, octamer-binding transcription factor 4, sex-determining region Y-box 2

## Abstract

**Background and Aim:**

CD 117 (c-KIT) internal tandem duplication (ITD), octamer-binding transcription factor 4 (Oct-4), and sex-determining region Y-box 2 (Sox-2) may govern the oncogenicity and aggressiveness of canine cutaneous mast cell tumor (MCT) in the crossbred dogs. Thus, a comprehension of this matter may help us establishing a novel platform to treat the disease in those dogs. However, evidence has lacked so far. Thus, this study aimed to survey CD 117 ITD, Oct-4, and Sox-2 expressions and their relations to the 2-tier grading in a group of Thai crossbreed dogs. The study was done using polymerase chain reaction (PCR), Reverse transcription PCR (RT-PCR), and immunohistochemistry.

**Materials and Methods:**

Thirty-three MCT specimens graded by the 2-tier histopathology grading were collected from the crossbred and purebred dogs. CD 117 ITD was detected by conventional PCR and immunohistochemistry. While, Oct-4 and Sox-2 expression levels were determined at the protein and mRNA levels by immunohistochemistry and RT-PCR, respectively. The expression magnitude of each parameter was then related to the grades and breeds.

**Results:**

About 60.61% of specimens were low grade, while 39.39% were high grade. CD 117 ITD was not detected in all specimens. A significant increase of Oct-4 expression was found in the high-grade, crossbred dogs. Meanwhile, Sox-2 expressions were increased both in the purebred and crossbred dogs with high-grade MCT.

**Conclusion:**

The study finding has indicated that the level of Sox-2 expression may be a useful tumorigenic and prognostic biomarker because it correlates to the 2-tier grades but not dog breeds.

## Introduction

Canine cutaneous mast cell tumor (MCT) is a highly prevalent, life-threatening skin malignancy in dogs [1]. Its biological behavior is apparently bizarre and somewhat unpredictable [2-4]. For example, the low grade is prone to relapse, although in a lower frequency when compared to the high grade. On the contrary, the high grade may not recur or metastasize as expected. As in other neoplasms, the oncogenesis of canine cutaneous MCT is still vague [1-4]. However, various studies have shown that MCT tumorigenesis has relied on such mutated genes that regulating mast cell proliferation and growth. Of these, mutations of the protooncogene *c-kit* encoding for type III tyrosine kinase receptor c-KIT (CD 117) have widely been investigated in the past several decades. This gene is essential for regulating the proliferation, differentiation, and degranulation of normal and cancerous mast cells [5]. Moreover, most studies have involved internal tandem duplication (ITD) in exon-11 [6-8]. Besides, recent evidence has indicated that canine cutaneous MCT contains a fraction of cancer stem-like cells. These putative cancer stem cells have unlimited self-renewal governed by a variety of embryonic transcription factors, including octamer-binding transcription factor 4 (Oct-4), sex-determining region Y-box 2 (Sox-2), and Nanog. Therefore, self-renewal has become the research of interest in MCT so far. For example, the early study from Webster *et al*. [9] in 2007 has demonstrated Oct-4 intranuclear immunopositivity in numerous MCT cells. In addition, the Pit-Oct-Unc embryonic transcription factor has been gained in other veterinary neoplastic species, such as canine mammary gland tumors, melanoma, and adenocarcinoma [10-13]. Furthermore, the Sox-2 intranuclear pattern has been reported in oligodendroglioma, melanoma, hemangiosarcoma, osteosarcoma, pulmonary carcinoma, hepatocellular carcinoma, complex mammary carcinoma, transitional cell carcinoma, and canine cutaneous MCT [14].

Apart from CD 117 and embryonic transcription factors, breed predisposition is another clinical parameter intensely evaluated worldwide in the past several years. Nevertheless, MCT clinical behavior and aggressiveness seem to be influenced by dog breeds [15]. A plethora of evidence has suggested that purebred dogs such as Boxers, Labrador Retrievers, Golden Retrievers, French Bulldogs, Dachshunds, and Shar-Pei are at risk of MCT [16-18]. However, little is known about crossbred dogs. They have ­usually been excluded from most studies due to the complexity of their inherited genes, causing result misinterpretation. Several studies have substantially shown the relevance of the crossbreed to canine cutaneous MCTs in several aspects. In one study, Horta *et al*. [4], have shown that 20% of mixed-breed dogs suffered from MCT. The other study from Śmiech *et al*. [19] in 2018 observed that 68% of crossbred dogs with MCT were low grade, while 32% were high grade.

Since the roles of exon-11 ITD, Oct-4, and Sox-2 on MCT formation in crossbred dogs have not been investigated yet. Moreover, over half of the petted dogs in Thailand are crossbred, and many are suffering from canine cutaneous MCTs. Accordingly, profiling and relating these tumorigenic parameters to the crossbred dogs and the 2-tier grading is essential. Thus, this study aimed to (1) survey the expressions of CD 117 ITD in exon-11, Oct-4, and Sox-2 at the molecular biology and immunohistochemistry levels in the crossbred dogs suffering from canine cutaneous MCT, and (2) relate each parameter to the 2-tier grading. We hypothesized that the result should reflect the relevance of one of the studied parameters to MCT tumorigenesis. Ultimately, this study should lead us to the way of disease elimination in the upcoming future.

## Materials and Methods

### Ethical approval

The research protocols were approved by the animal ethic committee of the Faculty of Veterinary Science, Chulalongkorn University (Ref No. 1631055), under the guidelines for the Care and Use of Experimental Animals, National Research Council of Thailand. All dog owners conceived the experiment and signed their consent forms for specimen usage.

### Study period and location

The study was conducted from August 2014-August 2020. The study was mainly conducted in the Oncology Clinic and Companion Animal Cancer Research Unit of the Faculty of Veterinary Science, Chulalongkorn University in Bangkok, Thailand, unless otherwise the other areas were declared.

### Animals and sample collection

Irrespective of gender, age, and location of the mass, 33 dogs with canine cutaneous MCT confirmed by fine-needle aspiration were selected from the oncology clinic. The mass of each was surgically removed in the operating theater and then halved. The first half was submitted to the pathology diagnostic center for the 2-tier grading and immunohistochemical profiling, while the other to the research unit for molecular profiling.

### Tissue handling and section preparation

The first tissue portion of each was fixed in 10% neutral buffered formalin for 5 days. Then, the preserved tissue was embedded with paraffin. Each formalin-fixed paraffin-embedded tissue was cut at 4 mm for preparing a series of four tissue slides. They were then deparaffinized, and one was stained with hematoxylin and eosin for 2-Tier grading. The remains were used for CD 117, Oct-4, and Sox-2 immunohistochemical profiling, as described below.

### 2-Tier grading

The 2-tier histopathologic grading system was performed on each specimen. Following the protocol and interpretative criteria previously described by Kiupel *et al*. [20] in 2011, the mitotic rate and nuclear features (i.e., karyomegaly, multinucleated cells, and bizarre) were investigated to determine the grade as low or high.

### Immunohistochemical profiling of CD 117, Oct-4, and Sox-2

Three immunohistochemistry sections were prepared in each case. The sections were washed with 1 × phosphate-buffered saline (PBS) twice and incubated with 1% (W/V) bovine serum albumin in 1 × PBS for 10 min to block non-specific proteins. The first section was incubated with CD 117 antibody at 4°C, in a dark and humidified chamber overnight. While, the second and third slides were incubated with Oct-4 and Sox-2 antibodies, respectively. The specific antibodies used are detailed in Table-1. Further, the sections were washed with 1× PBS twice and colorized with Envision^™^ kit (Dako, Denmark) using 3,3′-diaminobenzidine tetrahydrochloride as the substrate, for 45 min. The background noise was removed by washing the sections with 1× PBS. The nuclei were counterstained with Mayer’s hematoxylin. The membrane pattern of CD 117 was acquired with light microscopy. For embryonic transcription factors, MCT cells with intranuclear and nucleocytoplasmic patterns of Oct-4 and Sox-2 from five different areas were counted at the high-power field (40×), using Fiji version 1.53b (Image-J^®^, NIH, USA) using the deconvolution function. Canine testis was used as the positive control for both immunohistochemistry processes.

**Table-1:** List of antibodies used for immunohistochemical profiling.

Antibody	Dilution	Clone/Manufacturer
Rabbit-polyclonal anti-human CD 117 (c-KIT)	1:200	Dako, Denmark
Mouse monoclonal anti-human Oct-3/4 (Oct-4)	1:100	C-10, Clone SC-5279, Santa Cruz Biotechnology, USA
Mouse monoclonal anti-human Sox-2	1:100	Clone 560291 BD bioscience, USA

Oct-3=Octamer-binding transcription factor 4, Sox-2=Sex-determining region Y-box 2

### CD 117 mutation screening

Exon-11 ITD of *c-kit* was determined by conventional PCR with the specific primers as following.

F: 5′-CCA TGT ATG AAG TAC AGT GGA AG-3′

R: 5′-GTT CCC TAA AGT CAT TGT TAC ACG-3′

Stepwise, genomic DNA of each case was extracted and purified from 1 g of MCT sample using a commercial DNA isolation kit (Ultraclean^™^, Mobio, USA). The final volume of PCR cocktail was 25 μL, and it contained 12.5 μL of PCR master mix (AcessQuick^™^, Promega, USA), 2 μL of 10 mM forward and 2 μL of 10 mM reverse primers, 3 μL of DNA template, and 5.5 μL of DI water. PCR reaction was done with a thermocycler (G-Storm™, USA). The thermocycle was 95°C for 5 min for initiated DNA denaturation, 40 cycles of 95°C for 1 min, 57°C for 1 min and 95°C for 1 min for DNA amplification, and 95°C for 5 min for complete DNA elongation [21]. DI water was used as the negative control. The targeted amplicon was separated by 2% (W/V) agarose gel electrophoresis at 100 V for 30 min. The expected size of PCR product was 191 bp for the non-mutant exon-11 or 250 bp for exon-11 ITD, respectively.

### Reverse transcription-polymerase chain reaction (RT-PCR) profiling of Oct-4 and Sox-2

Briefly, the total RNA of each was extracted from 1 g of MCT tissue using a commercial RNA extract kit (PureLink™ RNA Mini Kit, USA) and stored at –80°C until used. cDNA library was constructed from 1 mg of total RNA using a commercial reverse transcription kit (High Capacity™, Applied Biosystems^®^, USA) under the manufacturer’s recommendation. The RT-PCR mixture consisted of 1.5 μL of cDNA, 12.5 μL of 2X PCR buffer (KOD FX Neo™, Toyobo^®^, Japan), 1 μL DNA polymerase (KOD FX Neo™, Toyobo^®^, Japan), 5 μL of 2 mM dNTPs (Thermo Fisher Scientific^®^, USA), 2.5 μL of forward primers, and 2.5 μL of reverse primers. The forward and reverse primers of Oct-4, Sox-2, and housekeeping gene β-actin and their production sizes are listed in Table-2 [22,23].

**Table-2:** List of Primers used in this study.

Gene	Primer sequences	Expected amplicon size (bp)	Reference
*Oct-4*	*F*: 5’-GAGTGAGAGGCAACCTGGAG-3’ *R*: 5’-GTGAAGTGAGGGCTCCC ATA-3’	437	[23]
*Sox-2*	*F*: 5’-AGTCTCAAGCGACGAAAAA-3’ *R*: 5’-GCA GA A G C CTCCTCTTGAA-3’	142	[23]
*β- actin*	*F*: 5’-TGTTGCCCTAGACTTAGACTTCGAGCA-3’ *R*: 5’-GGACCCAGGAAGGAAGGCT-3’	145	[22]

Oct 4=Octamer-binding transcription factor 4, Sox-2=Sex-determining region Y-box 2

The PCR was performed using GS-1 thermocycler (G-Strom™, Gene Technologies®, USA). Stepwise, the initial denaturation was set up at 95°C for 5 min. The thermocycling process consisted of 40 cycles of 95°C for 30 s, 55°C (β-actin) or 58°C (*Sox-2*) or 60°C (*Oct-4*) for 30 s, and 72°C for 30 s, followed by complete DNA extension at 72°C for 5 min. Then, 2% (W/V) agarose gel electrophoresis at 100 V for 30 min was performed to target the amplicons. The PCR products were further purified using QIAquick™ PCR purification kit (Qiagen, USA). They were then sequenced and blasted through NCBI blasting system (National Center for Biotechnology Information, USA).

### Statistical analysis

Statistical Package for the Social Sciences for Windows version 19 (IBM™, Armonk, New York, USA) was used for statistical analysis. The immunopositivity of Oct-4 and Sox-2 against 2-Tier grades were expressed in terms of mean and standard deviation. The comparison of expression levels to the grades between the purebred and crossbred was made using Mann–Whitney U-test, at p>0.05.

## Results

### MCT characterization

**Figure-1:**
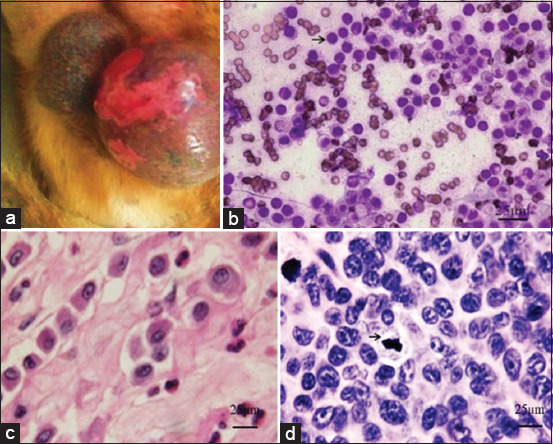
(a) An ulcerated skin plaque of the scrotum surrounded by inflammation and cutaneous edema in a case with poorly differentiated mast cell tumor (MCT). (b) Fine-needle aspiration showing many large round tumor cells with abundant cytoplasmic granules (Giemsa). (c) Low-grade MCT showing the uniform round to polygonal neoplastic cells containing abundant amphophilic cytoplasm with numerous fine basophilic intracytoplasmic granules. These tumor cells were supported by the collagenous stroma. (H&E). (d) High-grade MCT showing the pleomorphic neoplastic cells with anisokaryosis and numerous mitotic figures (H&E).

The neoplastic masses located on the different parts of the bodies. Well-differentiated MCT was solitary and not typically ulcerated. On the contrary, undifferentiated MCT was large and subjected to ulceration. In such circumstances, it might appear as an ulcerated skin plaque with moderate to severe inflammation and cutaneous edema of the surrounding tissues (Figure-1a). Fine-needle aspiration exhibited the cytologic feature of the round cell tumors. MCT cells were usually round with moderate cytoplasm containing fine metachromatic granules. Their nuclei were basophilic and round (Figure-1b). The cell size was varied from 12 μm to 20 μm, depending on degranulation.

### 2-Tier grading

Of 33 samples, 60.61% (n=20) of the dogs were defined as low-grade, while the remaining (39.39%, n=13) was the high-grade. Histopathological findings of the low grade showed the uniformity of the neoplastic cells. MCT cells were round and contained an abundance of intracytoplasmic granules. Their nuclei were round with prominent single nucleoli. The mitotic figure and multinucleated cells were rarely seen. The neoplastic cells were abundantly supported by collagenous stroma (Figure-1c). On the contrary to the high grade, MCT cells were pleomorphic. The cytoplasm and the cytoplasmic granules were usually scant. Multinucleated cells and the mitotic figure with bizarre nuclei were numerously seen throughout the fields, and the stromal support was less (Figure-1d).

### Immunohistochemical profiling

#### CD 117 immunohistochemistry

**Figure-2:**
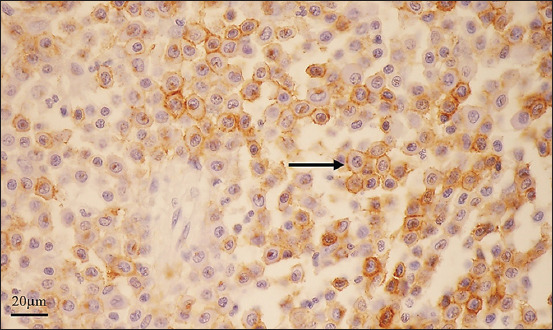
The membrane pattern of CD 117 immunohistochemistry in a specimen exhibits the immunopositivity on the cell membranes of such mast cell tumor cells (arrow).

The immunohistochemistry indicated the membranous pattern of CD 117 in all specimens but not all MCT cells. The immunopositivity was present on the cytoplasmic membranes (Figure-2). There was no difference in the expression among the breeds.

### Oct-4 immunohistochemistry

**Figure-3:**
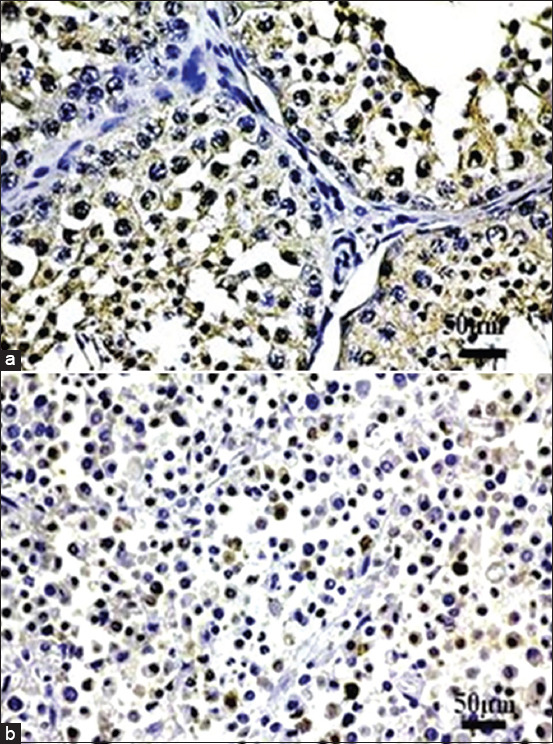
Octamer-binding transcription factor 4 immunohistochemistry, (a) In the testis, the immunopositivity is present in the nuclei of spermatocytes. (b) As in the testis, the immunopositivity exists in the nuclei of mast cell tumor cells.

Oct-4 intranuclear immunopositivity was present in all cases, as well as in the testis (Figure-3).

**Figure-4:**
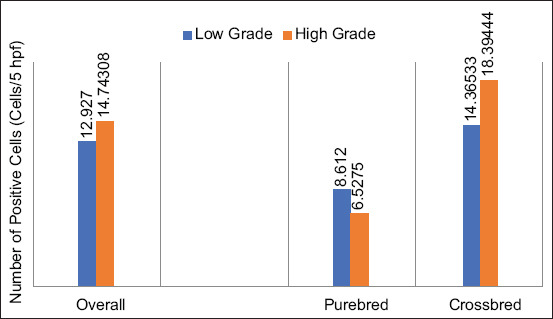
The histogram shows the tendency of octamer-binding transcription factor 4 expression under various conditions. In the overall panel, the expressions in the low grade and high grade are not different. The purebred dogs and crossbred dogs also have indifferently grade-related expressions. Notably, the expression is significantly increased in high-grade crossbred dogs.

On Image-J® analysis, regardless of breed predisposition, the overall feature suggested no difference in Oct-4 expression between both grades; 12.927±17.78135 cells/5 hpf in the low grade and 14.74308±13.25061 cells/5 hpf in the high grade. There was no grade-to-breed relation to the Oct-4 expression as well. The expression in the purebred, low-grade dogs was 8.612±5.51701, whereas the high grade was 6.5275±1.0054 cells/5 hpf. This similarity was also observed in the crossbred dogs. The expression in the low grade was 14.36533±20.2863 cells/5 hpf and 18.39444±14.63701 cells/5 hpf in the high grade, respectively. However, the expression in the high grade tended to increase slightly in the crossbred dogs, as compared to the pure breed (Figure-4).

### Sox-2 immunohistochemistry

**Figure-5:**
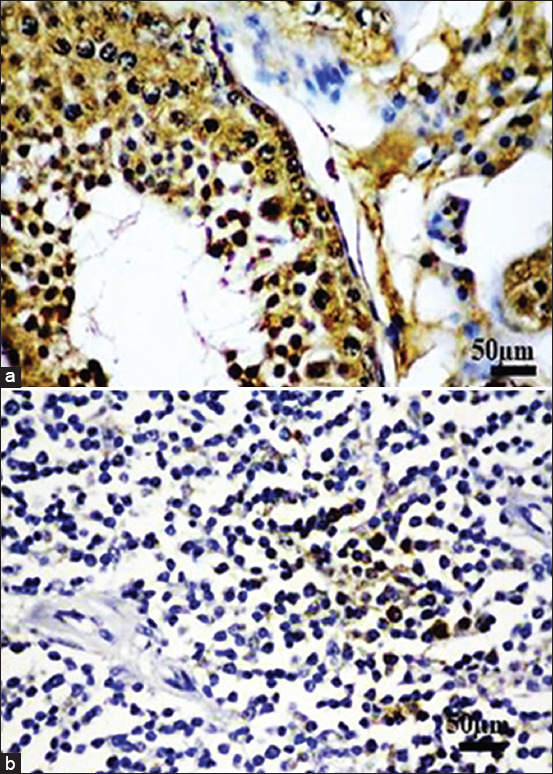
Immunohistochemical staining of sex-determining region Y-box 2 (Sox-2), (a) The immunopositivity is present in the nuclei of spermatocytes. (b) As in Oct-4, the nuclear immunolabeling is also present in MCT cells. This indicates the reactive Sox-2 in MCT cells.

Sox-2 was also present in the positive control and all MCT cases, as shown in Figure-5.

**Figure-6:**
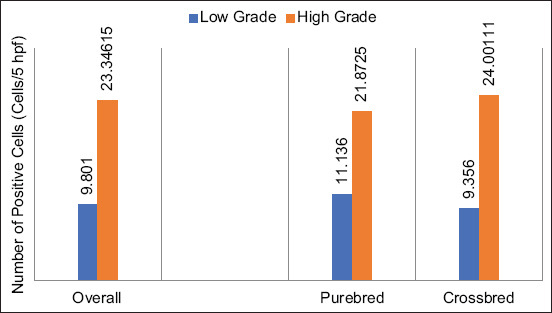
The histogram exhibits grade-dependent sex-determining region Y-box 2 (Sox-2) expressions. At the overall level (left), the expression in the low grade is significantly lower than in the high grade. This tendency is also present in all levels of the comparison. Sox-2 expressions of the low-grade mast cell tumor (MCT) in the purebred dogs and crossbred dogs are typically lower when compared to the high-grade MCT.

The overall picture showed that the levels of Sox-2 between the low and high grades were significantly different, from 9.801±12.88557 to 23.34615±12.57177 cells/5 hpf, respectively, (p>0.05). In the purebred, the immunopositivity in the high grade was higher than the low grade; 21.8725±11.1079 cells/5 and 11.136±11.10293 cells/5 hpf, respectively. The same trend was observed in the crossbred dogs, where the expression in the high grade was 2.6-fold higher than the low grade; 24.00111±13.75631 cells/5 hpf and 9.356±13.75744 cells/5 hpf, respectively. However, both purebred and crossbred dogs in the same MCT grade did not show any distinction in Sox-2 expression. Therefore, Sox-2 expression was grade-dependent and not breed-dependent (Figure-6).

### Molecular profiling

#### CD 117 mutation

**Figure-7:**
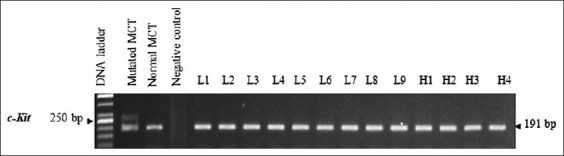
CD 117 mutation was not present in all specimens. All PCR products herein were 191 bp, when compared to the 250 bp mutant CD 117.

CD 117 ITD was grade-to-breed independent. The mutation was not observed in all specimens. There was not any distinction of mutation between grades and breeds. All PCR products were 191 bp (Figure-7). Amplicon sequencing and blasting also confirmed that the products were CD 117.

#### Expression of Oct-4

**Figure-8:**
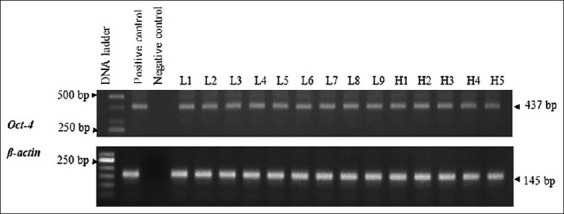
Reverse transcription-polymerase chain reaction exhibits the expression of octamer-binding transcription factor 4 embryonic transcription factor in all mast cell tumor specimens. The amplicons were 437 bp.

RT-PCR confirmed the existence of Oct-4 in MCT cells. The sequences of PCR products were compatible with canine Oct-4 based on the blasting. The amplicon size was 437 bp, and the expression was present in all specimens without breed and grade predilection (Figure-8).

#### Expression of Sox-2

**Figure-9:**
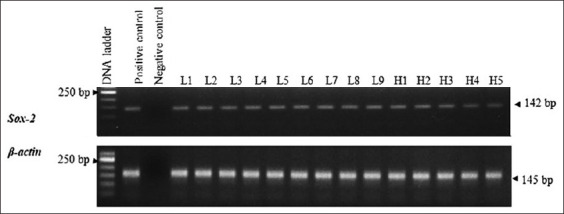
Sex-determining region Y-box 2 expression was present in all mast cell tumor specimens. The amplicon size was 142 bp. The expression was unrelated to 2-tier grades and breeds.

As in Oct-4, the expression of Sox-2 was confirmed by RT-PCR. The product size was 142 bp. NCBI blasting also confirmed the amplicons were canine Sox-2. The expression was found in all MCT samples (Figure-9). There was neither breed nor grade predisposition found.

## Discussion

Canine cutaneous MCT is one of the most common skin malignancies, with the prevalence accounted for 27% of all skin neoplasms [24-26]. The disease commonly takes place in middle-aged dogs and seems to be breed-predisposed. Unfortunately, traditional treatments are frequently ineffective since neoplastic behavior is conspicuously variable and hardly predictable. Although, some therapeutic agents such as the tyrosine kinase inhibitors can target several protooncogenes and decelerate the growth and recurrence. For example, toceranib phosphate selectively targets mutant CD 117 or masitinib mesylate inhibits stem cell factor/c-KIT pathway [27,28]. However, current evidence has indicated that this therapeutic setup is not sufficient for disease annihilation as well.

In a given neoplasm, it is composed of a small group of cancer stem-like cells. These dormant cells are responsible for cancer progression, distant propagation, relapse, and chemotherapy resistance [29-31]. Unfortunately, phenotyping of cancer stem-like cells is rarely successful since the morphometry of these cells cannot distinguish them from the non-cancer stem cell fractions. Cancer stem-like cells are immortal and highly evolved. They are capable of unlimitedly replenishing themselves. They are able to replace the lost or dead cancer cells with asymmetric cell division and pluripotent cell differentiation [32]. These functional characteristics are known as self-renewal, which is governed by numerous factors. Of these, Oct-4 and Sox-2 seem to be the key regulators [33]. The interplay between Oct-4 and Sox-2 can also form an intricate network for self-renewal modulation in several cancer stem-like cells [34-36]. Apart from the pioneer study of Webster *et al*. in 2007 [9], a plethora of evidence has recapitulated the presence of cancer stem-like cells in canine cutaneous MCT at the immunohistochemistry and mRNA levels[37].

In the present study, we have performed the molecular and immunohistochemical profiling of CD 117, Oct-4, and Sox-2 expressions in the purebred and crossbred dogs suffered from MCT. The results have provided interesting data that may increase our understanding of MCT tumorigenesis and progression. Three significant features have been defined in this study. First, the membrane pattern of CD 117 immunohistochemistry did not rely on the 2-tier grades. There was no ITD of exon 11 detected in all specimens. Second, at the mRNA level, the expression of Oct-4 was present in all samples. Neither grade nor breed predilection was found relating to Oct-4 immunolabeling because the number of the intranuclear positive cells was not different in both cases. Third, molecular evaluation has revealed that Sox-2 expression was also present in all specimens, regardless of grades or breeds. However, Sox-2 immunopositivity was significantly grade-dependent but not breed-predilected.

In line with the consequences, ITD of exon 11 did not directly drive MCT oncogenesis. Although, evidence has shown that the mutation is a significant factor for enhancing the aggressiveness and decreasing the survival time [1,4,38]. Irrespective of grading, the absence of the CD 117 ITD in the purebred and crossbred dogs did not mean that the disease was not aggressive or the survival time of the dogs was reduced, or even the prognosis was poor[38,39]. Accordingly, the ideal usage of exon-11 ITD as a tumorigenic indicator or prognosticator must be done carefully with caveats.

The presence of Oct-4 and Sox-2 in MCT has consistently implicated the existence of cancer stem-like cells with active self-renewal. However, Oct-4 expression alone is not an excellent tumorigenic or prognosticative biomarker. This study has demonstrated that Oct-4 expression at the mRNA or protein level was not typically different and was breed and grade-independent, even though the level was slightly higher in the high-grade MCT. Probably, Oct-4 indeed acts as an initiator to trigger the self-renewal and maintain the stem-like property of MCT cells.

Sox-2 immunohistochemical expression was likely an advanced tumorigenic or prognostic biomarker, although the molecular expression of Sox-2 was not significantly altered in all specimens. In contrast, Sox-2 immunolabeling was doubly increased in the high-grade MCT, regardless of breed predilections. This feature may infer the role of Sox-2 in taking over or augmenting Oct-4 self-renewal regulation. Furthermore, there is a propensity in which high-grade MCT will be aggressive.

According to the molecular and immunohistochemical surveys, both high-grade and low-grade crossbred dogs did not have the exon-11 ITD. However, unrestricted self-renewal regulated by Oct-4 and Sox-2 was present in a small fraction of MCT cells. While the level of Oct-4 immunopositivity was not varied, the Sox-2 level in the high-grade was 2.5-fold increased, compared to the low-grade. This similarity was described in the purebred dogs, but the level of Sox-2 immunolabeling was approximately two-fold in this case.

Taken together, we anticipate that the level of Sox-2 intranuclear immunolabeling may associate with MCT tumorigenesis and progression in the crossbred dogs. Therefore, this parameter may be a better predictive biomarker for MCT oncogenesis than the others. Ultimately, we recommend conducting a further study to verify the interaction of Oct-4 and Sox-2 in MCT crossbred dogs because the co-expression frequently enhances the aggressiveness and progression of some human neoplasms [40-42].

## Conclusion

In this study, we have immunohistochemically and molecularly profiled the expressions of CD 117, Oct-4, and Sox-2 in the crossbred dogs suffered from MCT, compared with the purebred. We have found that the level of intranuclear immunopositivity of Sox-2 may associate with grading and clinical behavior. This advantage indicates that this parameter may be useful as a tumorigenic and prognostic biomarker for MCT development. We also suggest performing a further study to determine the importance of concurrent Oct-4 and Sox-2 expressions. The future comprehension of these extents will lead the way to set up an effective target therapy for disease annihilation.

## Authors’ Contributions

AS, DK, and SM: Conceived and designed the experiment protocols. SM: Performed the ­experiments. SM, DK, and PP: Interpreted molecular biology results. SM, DK, AS, and KR: Analyzed immunohistochemistry data. DK and SM: Performed Image-J® analysis. SM and PT: Prepared the experimented reagents and specimens. DK, AS, and SM: drafted and revised the paper. DK contributes co-first authorship with SM and co-corresponding authorship with AS. All authors have carefully reviewed the manuscript and permitted it for publication.
